# Heart transplantation after acute myocardial infarction due to focal coronary Takayasu arteritis: a case report

**DOI:** 10.1093/ehjcr/ytad603

**Published:** 2023-11-30

**Authors:** Tor Skibsted Clemmensen, Christina Stilling, Sanne Bøjet Larsen, Hans Eiskjær

**Affiliations:** Department of Cardiology, Aarhus University Hospital, Palle Juul-Jensens Boulevard 99, 8200 Aarhus, Denmark; Department of Pathology, Aarhus University Hospital, Palle Juul-Jensens Boulevard 99, 8200 Aarhus, Denmark; Department of Cardiology, Aarhus University Hospital, Palle Juul-Jensens Boulevard 99, 8200 Aarhus, Denmark; Department of Cardiology, Aarhus University Hospital, Palle Juul-Jensens Boulevard 99, 8200 Aarhus, Denmark

**Keywords:** Case report, Acute heart failure, Acute myocardial infarction, Cardiogenic shock, Cardiopulmonary support, Heart transplantation, Takayasu arteritis

## Abstract

**Background:**

Takayasu arteritis is a chronic vasculitis of unknown aetiology primarily affecting medium to large arteries, particularly the aorta and arch vessels, and is predominantly seen in younger patients. Coronary artery involvement has been reported in 10–45% of autopsy cases, but isolated coronary Takayasu arteritis is extremely rare.

**Case summary:**

This case report describes the course of a previously healthy 22-year-old woman who suffered an acute myocardial infarction complicated by cardiogenic shock requiring temporary mechanical support subsequently urgent heart transplantation. The patient was bridged to transplant by the use of veno-arterial extracorporeal membrane oxygenation (VA-ECMO). The explanted heart showed evidence of Takayasu arteritis in the left coronary artery.

**Discussion:**

The case illustrates the importance of VA-ECMO treatment for cardiogenic shock, the importance of the Scandiatransplant collaboration for urgent organ allocation and the diagnostic difficulties associated with Takayasu arteritis.

**Learning points**

- Veno-arterial extracorporeal membrane oxygenation treatment can be lifesaving and should be considered prior diagnostic evaluation in selected patients with cardiogenic shock of unknown aetiology.
- The Scandiatransplant collaboration optimizes the donor organ utility and enables the opportunity of urgent heart transplantation.
- The Takayasu arteritis classification from the American College of Rheumatology falls short in diagnosing patients in the early disease stage when only a single arterial territory is affected.

## Introduction

Takayasu arteritis (TA) is a chronic vasculitis of unknown aetiology affecting medium to large arteries in younger patients.^1^ Acute myocardial infarction is reported in around 3.4% of TA patients but is rarely the first manifestation of the entity.^2^ This case describes the clinical course of a young woman suffering a TA-induced acute myocardial infarction and subsequently development of severe heart failure with the need of urgent heart transplantation. The case illustrates the importance of veno-arterial extracorporeal membrane oxygenation (VA-ECMO) treatment for cardiogenic shock, the Scandiatransplant collaboration significance for urgent organ allocation, and the diagnostic difficulties associated with TA.

## Summary figure

**Figure:**
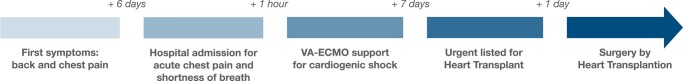


## Case presentation

A 22-year-old patient was admitted due to 6 days of back and chest pain. On the day of hospitalization, the pain was in progression and accompanied by shortness of breath and dizziness. The patient was previously healthy and free of medication and had no family history of heart disease.

**Figure 1:**
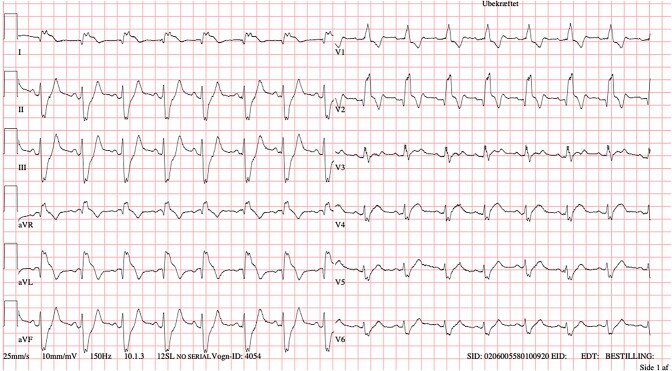
Acute electrocardiogram. Please note the right bundle branch block, ST-segment elevations in I, aVR, and aVL and the ST-segment depressions in II, III, and aVF.

**Figure 2:**
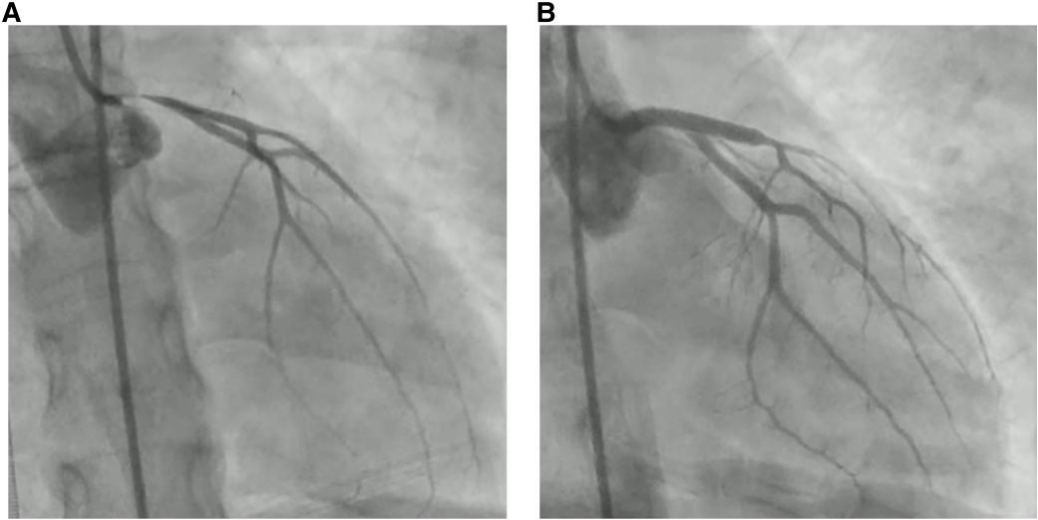
Coronary angiogram before (*A*) and after (*B*) percutaneous intervention.

Upon hospitalization, the patient was in shock with a blood pressure of 70/40 mmHg, heart rate of 105 b.p.m., and serum lactate of 7.9 mmol/L. Clinically, she was cold and dry but without pulmonary congestion. She was awake and had no fever. An acute electrocardiogram revealed sinus rhythm with right bundle branch block and ST-elevations in the anterolateral leads and ST-depressions in the inferior leads (*Figure 1*). An acute echocardiography revealed normal chamber sizes, no valve disease but severely reduced left ventricular ejection fraction (LVEF) < 10% and markedly reduced velocity–time integral (VTI) < 10 cm in the LV outflow tract confirming the cardiogenic shock suspicion (see Supplementary material online, *Video S1*). Subsequently, the in-hospital VA-ECMO team was summoned, and the patient transferred to the cardiac laboratory. During the transfer, the patient was in a near cardiac arrest stage needing repeated intravenous adrenalin administration. Venous and arterial cannulas were placed in the femoral vessels, and the ECMO circle was running within an hour after hospitalization. The patient immediately stabilized and a coronary angiogram revealed normal right coronary but sub-occluded left main coronary. Generally, the left-sided coronaries were very vasospastic (*Figure 2A*). Percutaneous intervention with stent implantation in the left main was performed uncomplicated (*Figure 2B*).

The patient was transferred to the intensive care unit where blood samples showed normal C-reactive protein, yet the white blood cells were increased to 25 × 10^9^/L. We noted significant myocardial necrosis with troponin I rising from 3734 to 409 590 ng/L. Serum lactate normalized, while renal and liver function remained preserved. Weaning attempts were performed on inotrope support on Days 4, 5, and 6 but unsuccessfully. On Day 7, we again attempted weaning during the administration of norepinephrine and milrinone but LVEF remained <20% and VTI <10 cm/s, mixed venous saturation dropped from 65% to 45%, and mean pulmonary arterial pressure increased from 19 to 30 mmHg with simultaneous drop in systemic pressure and need of high-dose norepinephrine. At this point, myocardial recovery seemed unlikely and the LV was still non-dilated with an end-diastolic diameter of <4 cm. A computed tomography scan in arterial and venous phase was normal, the patient had no HLA antibodies, and blood samples did not reveal any barriers for heart transplantation. Thus, the patient was activated on the urgent list in Scandiatransplant for heart transplantation, and after 4 h on the list, we received a donor call. The patient was successfully transplanted on Day 8 after hospitalization.

**Figure 3:**
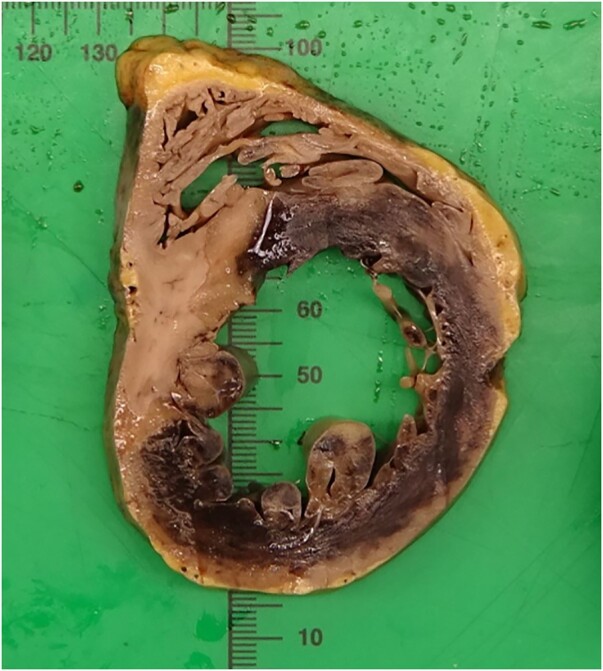
Short-axis section through the ventricles shows transmural infarction in left coronary artery territory distribution.

**Figure 4:**
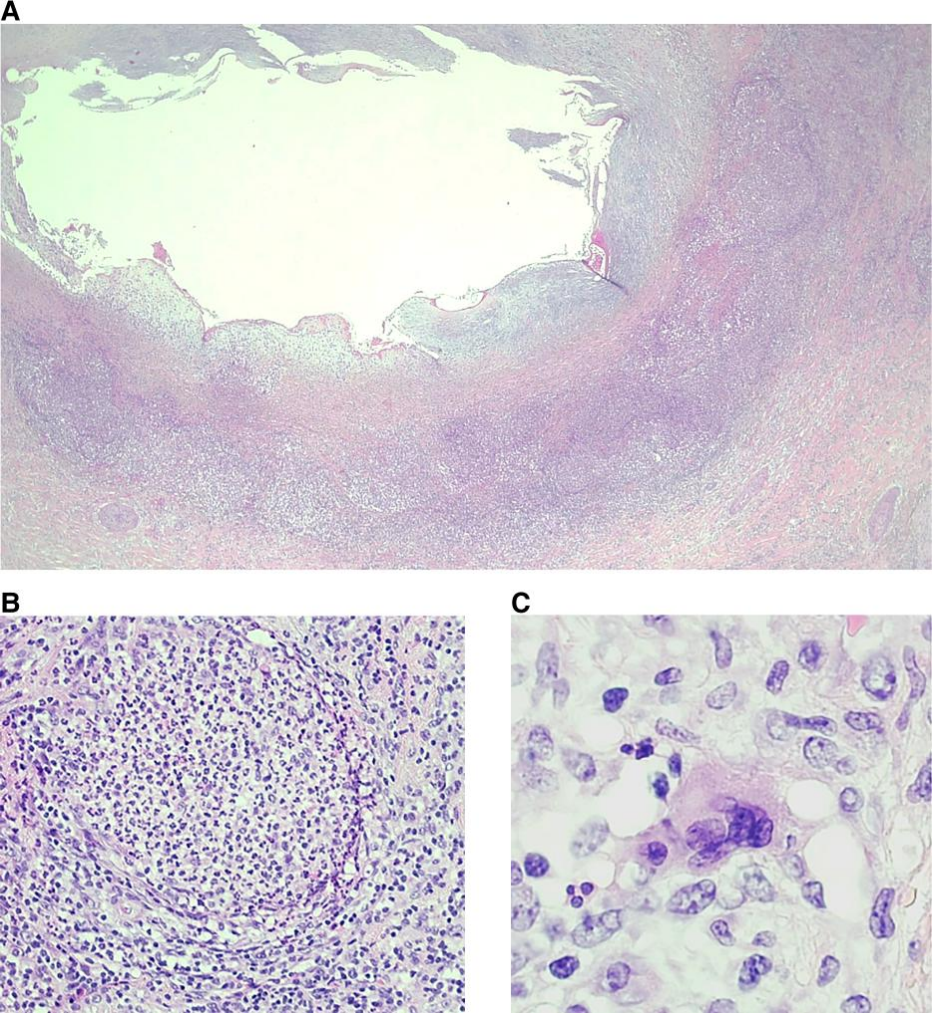
Haematoxylin and eosin stains: left coronary artery (*A*) shows acute phase changes of Takayasu disease with abundant lymphohistiocytic inflammation and microabscess-like necrosis (*B*) and fibrosis predominantly in adventitia. Few giant cells are singly distributed (*C*).

The explanted heart was sent for pathological examination. Gross photograph of the short-axis section through the ventricles revealed massive myocardial damage in the left coronary artery territory (*Figure 3*). Microscopy of the left coronary ostia revealed TA manifestations with intimal hyperplasia, abundant lymphohistiocytic inflammation, microabscess-like necrosis and fibrosis, the latter findings predominantly in adventitia. Also, a few singly distributed giant cells were noted (*Figure 4A–C*).

The patient has remained rejection free during the first 6-month follow-up. No fluorodeoxyglucose-positron emission tomography (FDG-PET) scan was performed as the results would likely be affected by ECMO cannulation and heart transplantation. Furthermore, the patient received immunosuppression following transplantation comprising corticosteroids, mycophenolate mofetil and tacrolimus, which would likely minimize the TA activity. The initial corticosteroid dose was intravenous methylprednisolone 500 mg for 3 days followed by oral prednisolone 2.5 mg/kg/day with a stepped reduction of 2.5 mg/week to a maintenance level of 7.5 mg/day. The patient has been tested negative for anti-neutrophil cytoplasmic antibodies, myeloperoxidase-AB, and proteinase-3-Ab.

A CT scan of the large arteries performed 3 months after transplantation showed moderate stenotic lesions of the right external iliac artery and occlusion of the left common femoral artery. However, these lesions could be explained by cannulation and sheath placement. The patient has not had any lower limb ischaemia symptoms.

## Discussion

###  

#### Veno-arterial extracorporeal membrane oxygenation for cardiogenic shock

Veno-arterial extracorporeal membrane oxygenation is increasingly used as a bridge to recovery or more advanced therapy in cardiogenic shock patients. While routine use of VA-ECMO in cardiogenic shock has not been demonstrated to be superior to initially conservative therapy,^3^ the patient in our case was in a near cardiac arrest stage when put on VA-ECMO. The severe myocardial damage and poor LV function implicate that conservative therapy would not have been sufficient. Thus, the early VA-ECMO use in our patient was lifesaving and served to maintain sufficient blood supply to vital organs, i.e. brain, liver, and kidneys. This was of utmost importance as the preserved end-organ function kept the window for heart transplantation open.

#### Scandiatransplant collaboration

The donor shortage makes optimal organ allocation systems necessary. In the Scandiatransplant countries, organs for heart transplantation are allocated to each participating centre. To ensure shorter waiting time for critically ill patients, the Scandiatransplant urgent heart allocation system had been serving to give supranational priority to patients considered urgent. Adult patients on temporary mechanical circulatory support and children on intravenous inotrope support can be listed as urgent. Results from the Scandiatransplant indicate that long-term survival in patients transplanted bridged by temporary mechanical circulatory support is excellent^4^ and the present case illustrates the effectiveness of the urgent heart allocation system.

#### Takayasu arteritis

Takayasu arteritis is a chronic vasculitis of unknown aetiology primarily affecting medium to large arteries, particularly the aorta and arch vessels.^1^ Adolescent girls and women in their second and third decades of life are most often affected.^1,5^ The diagnosis is based on clinical ischaemia manifestations from the affected organs in combination with imaging findings confirming vasculitis in arterial territories.^1^ The present case illustrates that the TA classification, from the American College of Rheumatology, falls short in diagnosing patients in the early disease stage when only a single arterial territory is affected. In this case, only the left main coronary was affected since CT scan of all other medium and large arteries initially did not reveal affection. Thus, the patient only scored 4/15 points in the classification criteria for TA, which is not diagnostic for the entity. Furthermore, the patient had no fever or elevated C-reactive protein indicating systemic inflammation. Thus, TA would not have been diagnosed if the patient had not undergone transplantation.

Isolated coronary TA has been described in the literature^6^ and coronary artery involvement has been reported in 10–45% of autopsy cases.^7^ Therefore, further assessment may be relevant in younger patients with angina or myocardial infarctions without significant risk factors. In such patients, evaluation with intracoronary imaging^6^ or FDG-PET^8^ could prove beneficial for early TA diagnosis.

The authors confirm that written consent for the submission and publication of this case report, including images and associated text, has been obtained from the patient in line with COPE guidance.

## Supplementary Material

ytad603_Supplementary_Data

## Data Availability

No new data were generated or analysed in support of this case presentation.

## Lead author biography

Tor Skibsted Clemmensen is a specialist in Cardiology. He completed his training at Aarhus University Hospital in 2022. His fields of interests are heart failure, heart transplantation, and cardiac imaging.

## Supplementary material

**Consent:** The patient provided oral and written consent for this publication.

**Funding:** The authors have not received any funding.
