# Supplementary figures and images for: Heart transplantation after acute myocardial infarction due to focal coronary Takayasu arteritis: a case report
Source: Eur Heart J Case Rep. 2023 Nov 30;7(12):ytad603. doi: 10.1093/ehjcr/ytad603 (PMC10714894; doi:10.1093/ehjcr/ytad603)

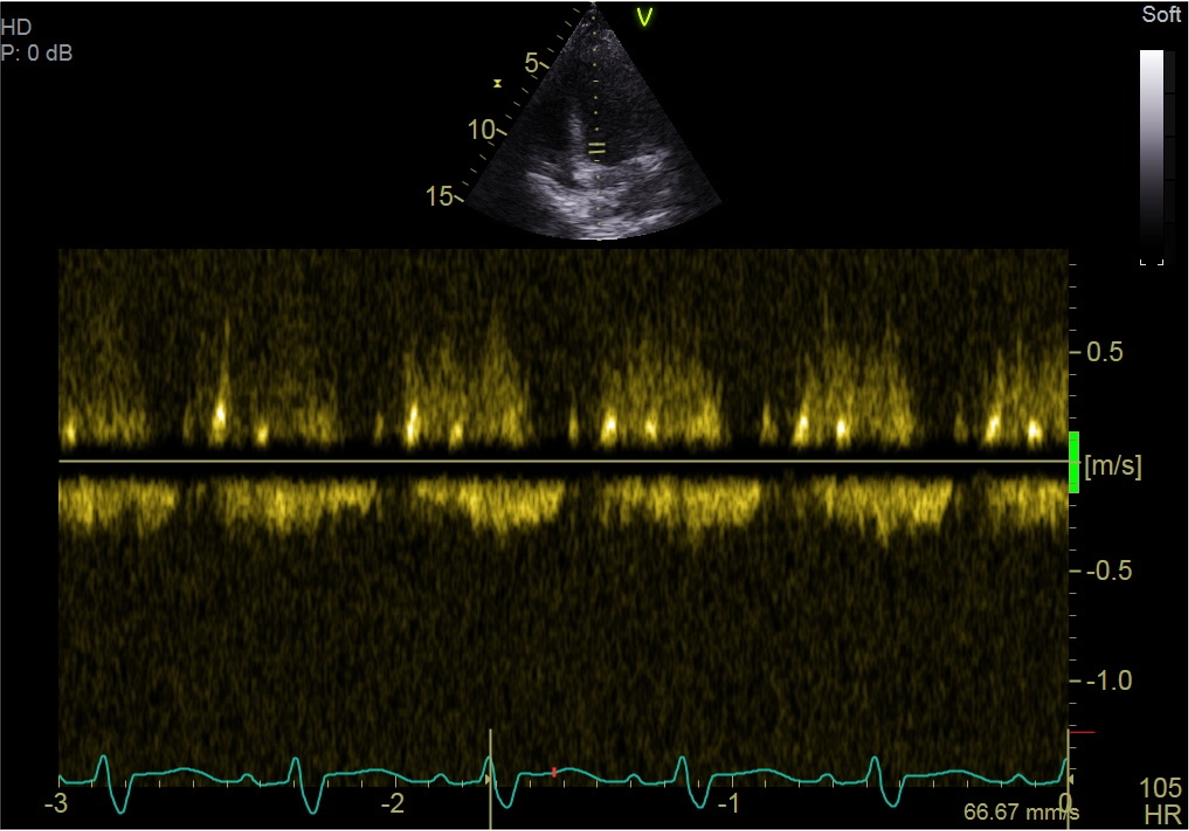

Supplement: ytad603_Supplementary_Data [file ytad603_supplementary_data.zip › LVOT_VTI.png]
